# Analysis of anthropometrics and mechanomyography signals as forearm flexion, pronation and supination torque predictors

**DOI:** 10.1038/s41598-022-20223-6

**Published:** 2022-09-27

**Authors:** Irsa Talib, Kenneth Sundaraj, Jawad Hussain, Chee Kiang Lam, Zeshan Ahmad

**Affiliations:** 1 University of Management and Technology, Lahore, Pakistan; 2 Universiti Teknikal Malaysia Melaka, Malacca, Malaysia; 3 Riphah International University, Lahore Campus, Lahore, Pakistan; 4 Universiti Malaysia Perlis, Arau, Malaysia

**Keywords:** Physiology, Engineering

## Abstract

This study aimed to analyze anthropometrics and mechanomyography (MMG) signals as forearm flexion, pronation, and supination torque predictors. 25 young, healthy, male participants performed isometric forearm flexion, pronation, and supination tasks from 20 to 100% maximal voluntary isometric contraction (MVIC) while maintaining 90° at the elbow joint. Nine anthropometric measures were recorded, and MMG signals from the biceps brachii (BB), brachialis (BRA), and brachioradialis (BRD) muscles were digitally acquired using triaxial accelerometers. These were then correlated with torque values. Significant positive correlations were found for arm circumference (CA) and MMG root mean square (RMS) values with flexion torque. Flexion torque might be predicted using CA (*r* = 0.426–0.575), a pseudo for muscle size while MMG_RMS_ (*r* = 0.441), an indication of muscle activation.

## Introduction

Mechanomyography (MMG) is a non-invasive technique for recording low-frequency oscillations in active skeletal muscle fibers^1^. MMG offers notable benefits, which include but not limited to, minimal skin preparation, negligible effect of skin impedance, and lower susceptibility to external noise^2,3^. Another benefit, which makes this technique more suitable than its electrical counterpart, surface electromyography (sEMG), is the information related to muscle contractile properties carried in the signal’s frequency content^4^.

The sEMG is a well-developed technique that has been applied in various areas related to muscle physiology including muscle assessment, prosthetics and exoskeleton development, and fatigue analysis^5^. These studies reveal that sEMG signals are found to be sensitive to exercise intensity^6^, speed variation^7^, and cognitive behavior^8^, among others. While MMG has been only recently researched for some of the above-mentioned areas, its results have been found promising and its application is gaining traction. The ability of MMG to quantify muscle functions using non-invasive means allows its potential usage for clinical applications^5^. It has been shown that contractile properties of muscles, which are directly related to the composition and fiber type of muscles, can be better estimated by using MMG frequency parameters. Furthermore, advanced multi-domain applications, such as neuromuscular functions, have also been characterized by MMG, like sEMG^9^.

A variety of transducers have been used to sense MMG signals. When the muscle is contracted, either through voluntary or simulated motor unit activity, dimensional changes occur within muscle fibers resulting in mechanical vibrations or oscillations^9^. The oscillatory effects of these contractions travel to the skin surface and are captured by MMG transducers. To get the most optimal MMG signal, muscle mass and sensor weight are the key considerations^10^. Several types of transducers including accelerometers, piezoelectric contact sensors, condenser microphones, and laser displacement sensors have been considered for acquiring MMG signals. As the transducer records surface oscillations on the skin, the risk of potential disturbances is quite high, hence lightweight MEMS accelerometers can be considered one of the best options for MMG signal acquisition^10^.

Torque estimation is one of the most important research areas in muscle assessment studies, specifically in those cases where it is difficult to estimate torque using direct methods or during activities that involve gross movements. There is evidence from the literature on the application of MMG as a tool for muscle torque estimation^9^. In one research study, time and frequency domain parameters of MMG signals have been plotted against torque produced by elbow flexor muscles^11^. It was found that time and frequency domains of MMG signal may reflect the dissimilarity in motor control startegies that regulate isometric and dynamic torque. Another work concluded that MMG can serve as a suitable approach for the estimation of muscle torque under those conditions where direct measurement of torque is not feasible, such as for sustained contractions under fatiguing conditions^12^. We believe the findings pertaining to torque estimation have the potential to benefit the research community working in the design and control of prosthetics. Further, these findings may also be useful in predicting overuse injury development in workplace settings that use repetitive joint maneuvers or high torque tasks^13^.

The elbow flexion movement is contributed by the three elbow joint flexors namely biceps brachii (BB), brachialis (BRA), and brachioradialis (BRD)^14^. All three muscles are considered in this study for the acquisition of MMG signals during sustained isometric forearm flexion, pronation, and supination tasks. The supination and pronation postures of the forearm are related to the BB and the BRA muscles^15^. The BB can be further subdivided into two muscle–tendon units. The lateral area of the BB and BRA is related to the pronation posture, and the medial area of the BB is related to the supination posture.

The ability of the forearm to produce muscle torque about the elbow joint is an important measure to gauge the ability to perform related workplace tasks. This outcome depends on both muscle activation levels and muscle cross-sectional area^13^. Suffice to say, anthropometric measures were observed to be predictive of elbow flexion strength^16^. Researchers found that forearm circumference and sEMG amplitude were observed to influence wrist torque^13^. Although, our previous study suggested MMG_RMS_ to be invariant to anthropometrics^17^, the analysis of anthropometrics and MMG signals as torque predictors is still in its infancy stage, and much remains to be uncovered. This paper aims to analyze anthropometrics and MMG signal parameters as predictors of forearm flexion, pronation, and supination torques. It was hypothesized that anthropometrics and MMG signal activation would exhibit positive correlations which could improve the estimation of torque in elbow flexor muscles.

## Methods

### MMG sensor and signal recording

**Figure 1:**
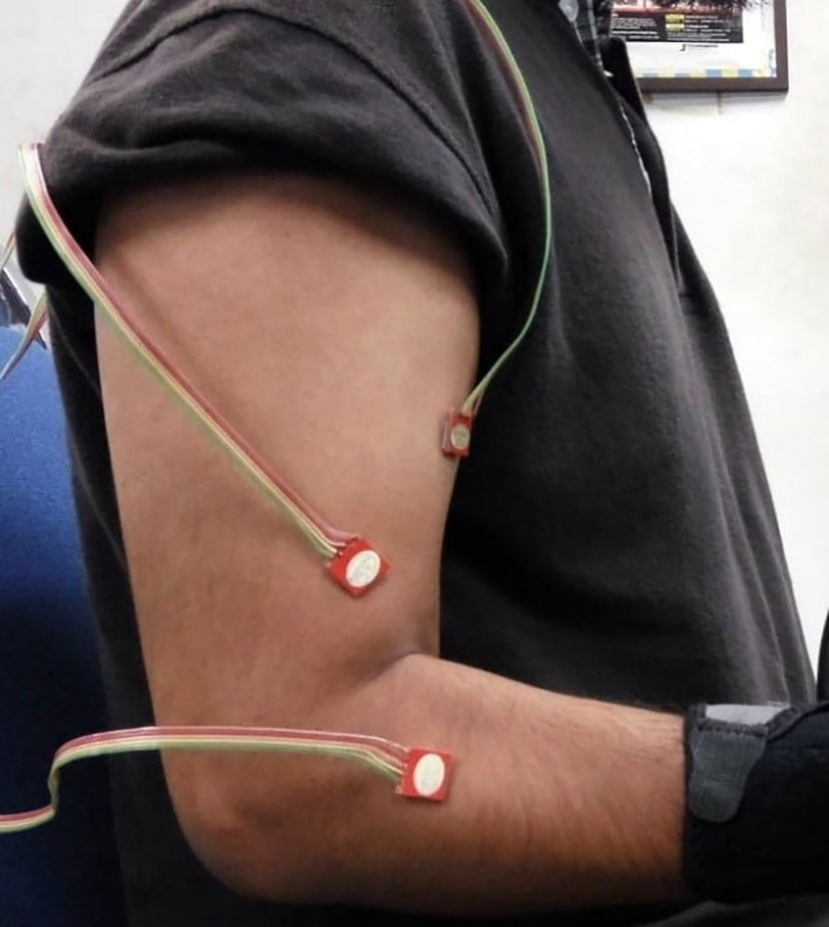
Placement of accelerometers on three elbow flexor muscles.

MMG signals were collected using three triaxial accelerometers (ADXL335, Analogue Devices, USA; full-scale range =  ± 3 g; typical frequency response = 0.5–500 Hz; sensitivity = 330 mVg^-1^; size = 15 mm × 15 mm × 1.5 mm; weight < 1.5 g). The design of experiment and procedures followed, were similar to our previous studies^1,17,18^. During the flexion task, accelerometers were placed on muscle bellies and attached using double-sided adhesive tape. This was done with the forearm in the supinated position and flexed at 90°. For the pronation and supination tasks, accelerometers were placed with the forearm in the neutral position and flexed at 90° (Fig. 1). The anatomical position of each muscle belly was determined according to^19^: BB—into the bulk of the muscle in the middle of the arm; BRA—two finger breadths proximal to the elbow crease along and just lateral to the tendon and bulk of the biceps; and BRD—midway between the biceps tendon and lateral epicondyle along the flexor crease. The *x*, *y*, and *z* axes of each accelerometer were positioned along the estimated longitudinal, lateral, and transverse directions of the muscle fibers, respectively. MMG signals recorded in the transverse axis of the accelerometer were utilized for further processing, as analysis in this direction is least influenced by crosstalk, an undesired effect^18^. The output from each of the sensors was connected to a data acquisition unit (NI cDAQ 9191 coupled with the NI 9205 module to yield an output at 16-bit resolution with CMRR of 100 dB, National Instruments, Austin, TX, USA), which was connected to a personal computer over Wi-Fi. The acquired MMG signals were digitally sampled at 1 kHz. Data acquisition and storage were performed using custom-made programs in LabVIEW™ (version 2016, National Instruments, Austin, TX, USA)^1,17,18,20^.

### Subjects

Twenty-five young, healthy, untrained, and right-handed male subjects (mean ± SD: age = 25.523 ± 4.545 years, weight = 68.219 ± 9.658 kg, height = 167.523 ± 3.172 cm) with no history of neuromuscular injury participated in the experiment. Written informed consent was taken from the subjects. The study was approved by the local Medical Research and Ethics Committee (MREC), Ministry of Health, Malaysia, and adhered to the guidelines established by the Declaration of Helsinki. A medical officer was present on site to aid the researchers and handle any emergency.

### Recording of anthropometric measurements

Anthropometrics such as arm length (LA), arm circumference (CA), age, weight, height, BMI, and skin fold thickness (ST) were considered in this study as torque predictors. The recording of these anthropometrics commenced through a familiarization session. The familiarization session was initiated by conducting a short briefing session about the experiments followed by taking written consent from the participants. Subsequently, during the first round, personal details and anthropometric measurements were noted by the experimenter. Later, during the second round, the same anthropometric measurements of all the participants were noted again in another experiment with a gap of at least 24 h between both rounds. The experimenter who recorded the anthropometrics underwent training, under the supervision of a medical officer, to accurately collect more than 150 test readings before taking the actual measurements required for this study. The CA, LA, and ST were measured using the same procedures as described in our previous study^17^. The details of these anthropometrics along with other parameters of all the subjects are given in Table 1.

**Table 1:** Anthropometric details of subjects.

Subject #	Age (Years)	Weight (kg)	Height (cm)	BMI (kgm^−2^)	LA (cm)	CA (cm)	ST-BB (mm)	ST-BRA (mm)	ST-BRD (mm)
1	34	82.6	167	29.617	35.5	34.5	12.8	15.6	12.8
2	32	65.5	161	25.269	36	29.8	10.4	14.4	14.4
3	25	54.8	167	19.649	36.5	32.8	8.4	10	9.6
4	32	91.9	183	27.442	41	32.8	10.8	14.4	14.8
5	21	56.1	157	22.760	35	28.5	8.8	9.2	8.8
6	22	73.4	168	26.006	38	35	8	10.4	10
7	23	56.5	169	19.782	37	26.7	10.4	11.2	10
8	21	55.8	169	19.537	35.5	28.3	10.4	8.4	10.4
9	23	66.6	160	26.016	35	32.3	7.6	9.6	10.8
10	23	67.5	163	25.406	36	35.5	8	9.2	8.8
11	35	89.2	172	30.151	39.5	35.1	10.4	8.8	8.8
12	35	89.1	181	27.197	38	31.6	8.4	10.8	10.4
13	28	70.8	170	24.498	38.5	34.3	7.6	9.2	10
14	23	80.3	167	28.793	36.5	35.5	11.6	12.8	13.2
15	42	75.5	167	27.072	33.5	33.8	6.8	10	8.4
16	22	62	162	23.624	35	30	7.6	8.8	8.8
17	22	43.7	166	15.859	33	24.8	7.2	6.8	7.2
18	21	62.9	165	23.104	37	33	8.4	8	7.6
19	32	65.5	168	23.207	37	30.16	5.2	7.6	8
20	23	69.9	170	24.187	37.5	34.5	8	10.4	9.6
21	23	54.7	164	20.338	36	31.6	7.2	8.8	8.4
22	24	68.3	170	23.633	36	32.6	8	8.4	9.6
23	22	78.7	173	26.296	39	39.8	8.4	9.2	11.2
24	23	56.5	170	19.550	36.5	28.5	6.4	8	7.6

BMI, body mass index; LA, length of arm; CA, circumfernec of arm; ST, skin fold thickness; BB, biceps brachi; BRA, brachialis; BRD, brachioradialis.

### Determination of MVIC

Each participant completed the experiment in two sessions. In the first session, maximal voluntary isometric contractions (MVIC) were determined for all three isometric tasks. Three trials were performed for each task with a gap of 2 min between trials and 10 min between tasks. Each participant followed postural settings similar to those described in our previous study^17^.

The maximum weight (in the form of dumbbells) that a participant could isometrically sustain for 2–3 s in proper posture and forearm in a supinated position, was considered as MVIC for the flexion task. The participant held a wrist dynamometer (Baseline™ Evaluation Instruments, Fabrication Enterprises Inc., NY, USA) for MVIC determination in the pronation and supination tasks. The wrist was maintained at a neutral position and MVIC was determined as the maximum effort the participant could produce for 2–3 s of forearm pronation or supination. Only trials with variations less than ± 5% from the expected values were acceptable. Participants were strongly encouraged verbally to produce their maximum effort for MVIC determination in all three tasks.

### Submaximal to maximal tasks

In the second session, subjects performed warmups, followed by submaximal tasks using similar postural settings. Subjects were asked to perform three isometric tasks (flexion, pronation, and supination) at 20%, 40%, 60%, 80%, and 100% MVIC for at least 6 s, with an inter- and intra-task rest of at least 10 min and 2 min, respectively. However, our recordings show that for 100% MVIC, most of the participants could hold only for at most 4 s, which was nevertheless considered sufficient for data processing. All the trials per task were randomized in terms of levels of effort to avoid any risk of biasness in the data. Subjects were not informed of the order prior to the commencement of the task. Trials were repeated if the observed torque levels presented a deviation of ± 5% of the expected values. During the trials, proper posture, off-axis precautionary measures, maximum ± 5% variations, announcement of elapsed time, and verbal encouragement were observed. The generated signals were recorded on a personal computer for further analysis.

### Data analysis

The data stored on a personal computer were digitally bandpass filtered (4th-order Butterworth) at 5–100 Hz to obtain the MMG signals. Only those collected signals that provided a signal-to-noise ratio > 10 dB were used for further processing. The 2-s long MMG signals from the middle of each isometric contraction were then extracted to avoid transient phenomenon when the muscle passes from rest to activity and vice versa, as recommended by^21^. These segments were then used to quantify RMS.

### Statistical analysis

Statistical analyses were performed using IBM SPSS (version 20, IBM SPSS Statistics, NY, USA). Data normality was checked using Shapiro–Wilk’s test and the acquired data from the experiments was found to be normally distributed. Thus, parametric analyses were employed in all subsequent computations. Linear regression was performed to determine the estimated relationship of the torque values with RMS and anthropometric parameters. Pearson’s correlation coefficients (*r*) at a significance level of 0.05 were used to describe the correlation between the variables as follows: 0.00 to ± 0.30 = negligible; ± 0.30 to ± 0.50 = low; ± 0.50 to ± 0.70 = moderate; ± 0.70 to ± 0.90 = high; ± 0.90 to ± 1.00 = very high^22^.

## Results

Tables 2, 3, 4 summarize Pearson’s correlation coefficient (*r*), goodness of fit (*r*^2^), and root mean squared error (RMSE), for torque against the twelve anthropometric parameters and MMG_RMS_, during flexion, pronation, and supination tasks.

**Table 2:** Bivariate analysis of torque against anthropometrics and MMG_RMS_ during flexion task.

Parameters	20% MVC			40% MVC			60% MVC			80% MVC			100% MVC		
	*r*	*r*^2^	RMSE	*r*	*r*^2^	RMSE	*r*	*r*^*2*^	RMSE	*r*	*r*^*2*^	RMSE	*r*	*r*^*2*^	RMSE
LA	0.01	0.000	2.041	0.089	0.008	2.875	0.112	0.013	4.817	0.135	0.018	5.524	0.087	0.008	6.731
CA	0.276	0.076	1.962	**0.575**	0.331	2.361	**0.426**	0.181	4.385	**0.558**	0.311	4.626	**0.524**	0.275	5.755
Age	− 0.176	0.031	2.009	− 0.362	0.131	2.690	− 0.199	0.040	4.750	− 0.262	0.069	5.380	− 0.279	0.078	6.489
Weight	− 0.077	0.006	2.035	0.044	0.002	2.883	0.093	0.009	4.826	0.149	0.022	5.513	0.094	0.009	6.727
Height	− 0.211	0.045	1.995	− 0.172	0.030	2.843	− 0.069	0.005	4.835	− 0.053	0.003	5.567	− 0.118	0.014	6.710
BMI	0.018	0.000	2.041	0.152	0.023	2.852	0.146	0.021	2.041	0.210	0.044	5.451	0.176	0.031	6.652
ST BB	− 0.202	0.041	1.999	− 0.086	0.007	2.875	− 0.068	0.005	4.836	− 0.025	0.001	5.573	− 0.064	0.004	6.743
ST BRA	− 0.094	0.009	2.032	− 0.095	0.009	2.873	− 0.119	0.014	4.813	− 0.083	0.007	5.556	− 0.085	0.007	6.733
ST BRD	− 0.07	0.005	2.036	− 0.060	0.004	2.881	− 0.077	0.006	4.833	− 0.029	0.001	5.573	− 0.043	0.002	6.751
RMS BB	0.37	0.137	1.896	0.014	0.000	2.886	0.108	0.012	4.819	0.030	0.001	5.572	− 0.087	0.008	6.731
RMS BRA	0.386	0.149	1.883	0.000	0.000	2.886	0.014	0.000	4.847	− 0.213	0.045	5.447	− 0.227	0.052	6.581
RMS BRD	**0.441**	0.194	1.832	0.059	0.003	2.881	0.068	0.005	4.836	− 0.159	0.025	5.504	− 0.168	0.028	6.661

*Bold font indicates significant correlations.

**Table 3:** Bivariate analysis of torque against anthropometrics and MMG_RMS_ during pronation task.

Parameters	20% MVC			40% MVC			60% MVC			80% MVC			100% MVC		
	*r*	*r*^*2*^	RMSE	*r*	*r*^*2*^	RMSE	*r*	*r*^*2*^	RMSE	*r*	*r*^*2*^	RMSE	*r*	*r*^*2*^	RMSE
LA	0.144	0.021	3.492	0.165	0.027	7.064	0.170	0.029	10.545	0.167	0.028	13.882	0.170	0.029	17.423
CA	0.195	0.038	3.461	0.241	0.058	6.951	0.244	0.060	10.378	0.237	0.056	13.679	0.242	0.059	17.155
Age	− 0.175	0.031	3.475	− 0.293	0.086	6.848	− 0.291	0.085	10.238	− 0.290	0.084	13.475	− 0.287	0.082	16.936
Weight	0.092	0.009	3.514	0.113	0.013	7.116	0.121	0.015	10.622	0.123	0.015	13.973	0.122	0.015	17.548
Height	0.015	0.000	3.529	0.181	0.033	7.044	0.183	0.034	10.520	0.187	0.035	13.832	0.185	0.034	17.375
BMI	0.109	0.012	3.508	0.038	0.001	7.157	0.046	0.002	10.690	0.046	0.002	14.065	0.046	0.002	17.661
ST BB	− 0.057	0.003	3.523	0.183	0.034	7.041	0.186	0.035	10.514	0.187	0.035	13.832	0.184	0.034	17.378
ST BRA	0.026	0.001	3.528	− 0.027	0.001	7.159	− 0.031	0.001	10.696	− 0.025	0.001	14.076	− 0.026	0.001	17.674
ST BRD	− 0.001	0.000	3.529	0.149	0.022	7.082	0.147	0.022	10.585	0.155	0.024	13.910	0.151	0.023	17.477
RMS BB	− 0.01	0.000	3.529	0.100	0.010	7.126	− 0.063	0.004	10.680	− 0.045	0.002	14.066	0.039	0.002	17.667
RMS BRA	0.059	0.004	3.523	0.134	0.018	7.097	− 0.099	0.010	10.648	0.168	0.028	13.880	0.057	0.003	17.651
RMS BRD	− 0.017	0.000	3.529	0.241	0.058	6.951	0.054	0.003	10.685	0.206	0.042	13.778	0.294	0.086	16.899

*Bold font indicates significant correlations.

**Table 4:** Bivariate analysis of torque against anthropometrics and MMG_RMS_ during supination task.

Parameters	20% MVC			40% MVC			60% MVC			80% MVC			100% MVC		
	*r*	*r*^*2*^	RMSE	*r*	*r*^*2*^	RMSE	*r*	*r*^*2*^	RMSE	*r*	*r*^*2*^	RMSE	*r*	*r*^*2*^	RMSE
LA	− 0.122	0.015	2.521	− 0.101	0.010	5.253	− 0.086	0.007	7.871	− 0.032	0.001	10.564	− 0.094	0.009	13.132
CA	0.159	0.025	2.508	0.173	0.030	5.200	0.188	0.035	7.759	0.191	0.036	10.374	0.189	0.036	12.952
Age	0.002	0.000	2.540	0.017	0.000	5.279	0.018	0.000	7.899	0.039	0.002	10.561	0.024	0.001	13.186
Weight	0.151	0.023	2.511	0.175	0.031	5.199	0.185	0.034	7.764	0.236	0.056	10.270	0.190	0.036	12.950
Height	− 0.126	0.016	2.520	− 0.098	0.010	5.255	− 0.104	0.011	7.857	− 0.028	0.001	10.565	− 0.102	0.010	13.121
BMI	0.236	0.056	2.468	0.252	0.064	5.110	0.268	0.072	7.611	0.290	0.084	10.115	0.273	0.075	12.689
ST BB	0.214	0.046	2.481	0.199	0.040	5.174	0.204	0.042	7.734	0.232	0.054	10.281	0.216	0.047	12.879
ST BRA	− 0.025	0.001	2.539	− 0.050	0.003	5.273	− 0.041	0.002	7.893	0.008	0.000	10.569	− 0.028	0.001	13.185
ST BRD	0.155	0.024	2.509	0.137	0.019	5.230	0.142	0.020	7.820	0.198	0.039	10.360	0.147	0.022	13.047
RMS BB	− 0.011	0.000	2.540	0.212	0.045	5.160	− 0.040	0.002	7.894	− 0.101	0.010	10.515	− 0.264	0.070	12.722
RMS BRA	− 0.007	0.000	2.540	− 0.396	0.157	4.848	− 0.125	0.016	7.838	0.281	0.079	10.143	− 0.364	0.132	12.285
RMS BRD	− 0.088	0.008	2.530	− 0.111	0.012	5.247	0.009	0.000	7.900	− 0.017	0.000	10.567	− 0.054	0.003	13.171

*Bold font indicates significant correlations.

During all three isometric tasks at five different submaximal to maximal effort levels, the values of *r*, *r*^2^ and RMSE varied from − 0.396 to 0.575, 0.000 to 0.331, and 1.832 to 17.674 respectively. The value of *r* was during all three isometric tasks observed to vary from – 0.122 to 0.175 for LA, 0.159 to 0.575 for CA, − 0.175 to 0.039 for age, − 0.077 to 0.236 for weight, − 0.028 to 0.187 for height, 0.018 to 0.290 for BMI, − 0.086 to 0.232 for ST BB, − 0.119 to 0.026 for ST BRA, − 0.077 to 0.198 for ST BRD, − 0.010 to 0.370 for RMS BB, − 0.007 to 0.386 for RMS BRA and − 0.017 to 0.441 for RMS BRD. The overall range of *r* for isometric tasks was noted as − 0.362 to 0.575, − 0.293 to 0.294, and − 0.399 to 0.29 during isometric flexion, pronation, and supination tasks respectively.

**Figure 2:**
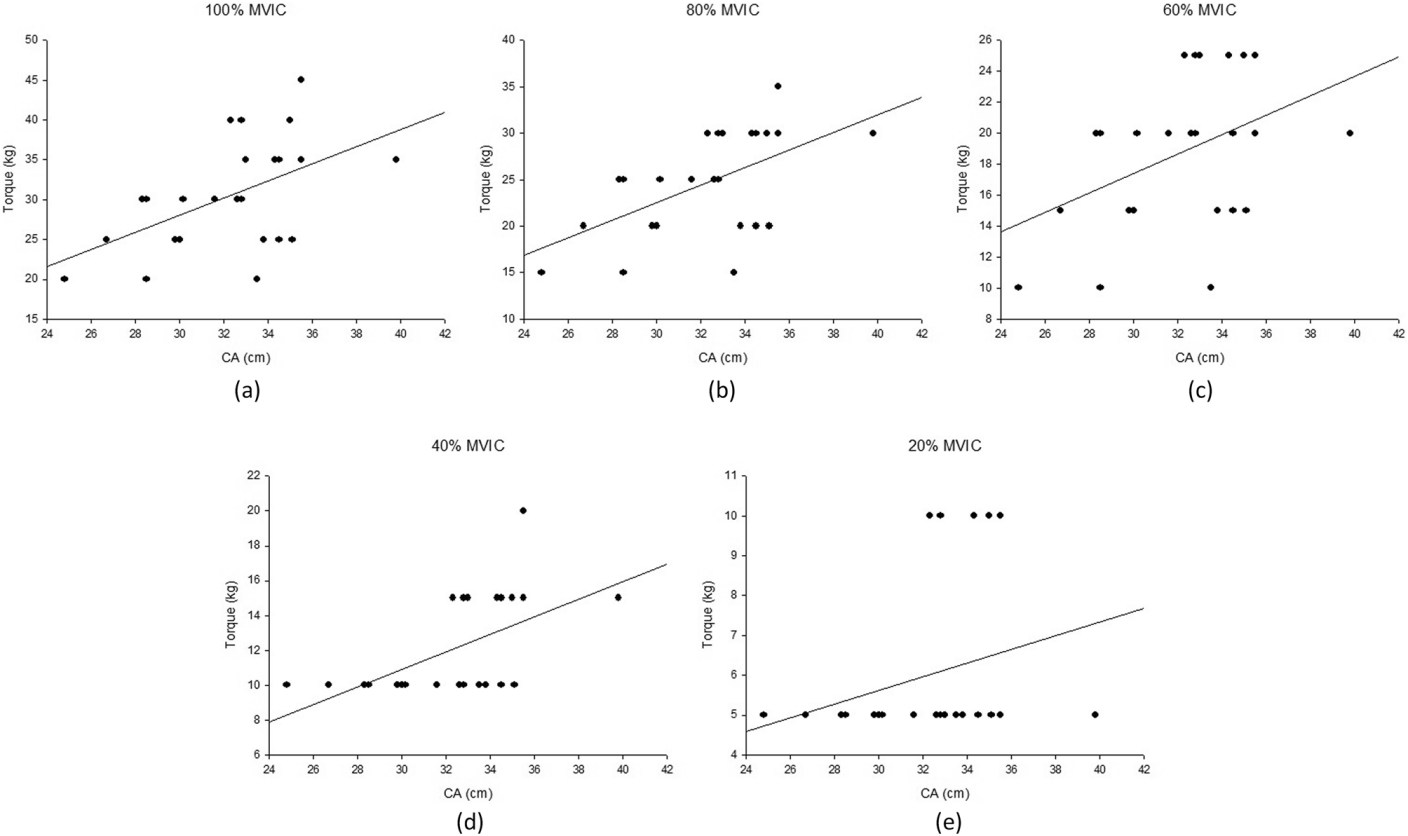
Scatter plots between torque and CA at (**a**) 100%, (**b**) 80%, (**c**) 60%, (**d**) 40% and (**e**) 20% MVIC during forearm flexion tasks.

Positive correlations were observed for torque with CA (*r* = 0.159 to 0.575) and torque with BMI (*r* = 0.018 to 0.290) during all three isometric tasks. Pearson's correlation of torque with CA were observed to be significant (*r* = 0.426 to 0.575, *r*^2^ = 0.181 to 0.331, RMSE = 2.361 to 5.755) during isometric flexion task at 40–100% MVIC. Scatter plots in Fig. 2 show the linear regression line for torque against CA. The values of *r* between torque and RMS was found significant (*r* = 0.441, *r*^2^ = 0.194, RMSE = 1.832) for BRD at 20% MVIC. The values of *r*^2^ varied from 0.000–0.331, 0.000–0.086, and 0.000–0.157 during isometric flexion, pronation, and supination tasks respectively. Significant and low positive correlations were observed for CA and RMS BRD against torque. It was observed from the scatter plots of CA against muscle torque that positive correlation was exhibited between the two parameters as indicated by their linear regression line. However, the points in the scatter plots for lower submaximal effort levels, specifically at 20 and 40% MVIC, were observed to be rather concentrated at a few torque values.

RMSE was observed to be increasing with ascending levels of effort from 20 to 100% MVIC in all three isometric tasks. RMSE was observed to range from 1.832–2.041, 2.361–2.886, 4.385–4.847, 4.626–5.573 and 6.489–6.751 at 20%, 40%, 60%, 80% and 100% MVIC respectively, during the isometric flexion task. Similarly, RMSE varied from 3.461–3.529, 6.848–7.157, 10.378–10.696, 13.679–14.076 and 16.936–17.674 at 20%, 40%, 60%, 80% and 100% MVIC respectively, during the isometric pronation task. Finally, RMSE ranged from 2.468–2.540, 5.11–5.279, 7.611–7.900, 10.115–10.569 and 12.689–13.186 at 20%, 40%, 60%, 80% and 100% MVIC, respectively during the isometric supination task.

## Discussion

From an ergonomics point of view, anthropometrics^23^ and strength capabilities^24^ play important roles in workplace design towards the prevention of injury, specifically in the case of workers performing repetitive tasks involving forearm pronation and supination, and elbow joint flexion. Thus, this study was designed to analyze anthropometrics and MMG signals as forearm flexion, pronation, and supination torque predictors. We hypothesized that a few anthropometrics and MMG signal parameters will serve as torque predictors among the elbow flexors. In order to test this hypothesis, correlations of torque, at five submaximal to maximal levels of effort, with nine anthropometrics, and MMG_RMS_ from the BB, BRA and BRD muscles were analyzed. Among the nine investigated anthropometrics, only CA observed the highest correlation with muscle torque during the isometric flexion task at 40% through 100% MVIC (*r* = 0.426–0.575). The maximum regression values (*r*^*2*^ = 0.181–0.331) observed during the submaximal to maximal isometric flexion task provided evidence of CA being an acceptable torque predictor. These findings support our hypothesis that there is substantial influence by the circumferential measurements of the arm and MMG_RMS_ on flexion torque.

Literature indicates that CA is a measure of muscle size and provides an indication of the amount of contractile tissue^25–27^. While this is true, the findings in this study reveal that CA is more influential in generating muscle torque during the elbow joint flexion tasks, as compared to the forearm pronation and supination tasks. Nevertheless, we do not deny that the participation of all three investigated muscles during the elbow joint flexion might be the reason CA appeared as a significant torque predictor. Significant Pearson’s correlation was observed between MMG_RMS_ from the BRD and joint torque (*r* = 0.441) during the elbow flexion task. Thus, we can conclude that the acquired MMG signal contains information on muscle activation, and the torque produced at the elbow joint is a result of that activation levels, similar to the conclusions obtained in^28^. This observation further strengthens our findings on anthropometrics, specifically on CA, to being a prominent torque predictor of the elbow flexion task.

Relationships between muscle size and torque generation have been used in musculoskeletal modeling studies to predict individual muscle forces. Our study observes CA having a weak to moderate positive correlation with muscle torque (*r* = 0.426–0.575). This finding concurs with similar correlations observed in other studies^29,30^. Further, moderate correlations were observed at four distinguished levels of effort (40%, 60%, 80% and 100% MVIC). We believe that this observation signifies that muscle torque prediction would not be limited to only low or high levels of effort. Musculoskeletal modeling is conducted, more than often, by purposefully determining individual muscle forces and not the maximum joint force. Thus, having information on the correlation between torque and CA, as revealed by our study, could provide an alternative way towards efficient modeling by being able to estimate individual muscle contributions. In addition, insights into how torque is correlated to anthropometrics and MMG signals have the potential to improve the control and design of prosthetics.

The contribution of muscle activation toward muscle torque prediction has been assessed through sEMG RMS in the past^13,16^. In this work, MMG_RMS_ from the BRD and muscle torque during the elbow flexion task were observed to have significant correlation (*r* = 0.441). We believe this correlation, between MMG signal parameters and torque prediction, could be largely improved by including an analysis of the antagonistic coactivation muscles. In addition, a digital dynamometer may be utilized during the isometric flexion task to obtain torque measurements at higher resolutions. This would certainly improve the scatter of acquired data, specifically at the lower effort levels (20 and 40% MVIC), as shown in the regression lines drawn in Fig. 2. The observed accumulation of data points in the scatter plots of low effort levels might be due to the low resolution in the measured torque values. Hence, a protocol designed with a different and sophisticated experimental setup, including fatiguing contractions^7^, agonistic and antagonistic muscle coactivations^5^, and participants from both genders, might add more clarity to torque prediction through anthropometrics and MMG signal.

## Conclusion

Significant positive correlations were found for flexion torque with CA (*r* = 0.426–0.575) and MMG_RMS_ (*r* = 0.441). This observation leads us to conclude that muscle torque is a predictor or pseudo for muscle size and activation levels measured through CA and MMG_RMS_ respectively. These findings on torque correlated with anthropometrics and MMG signals might be useful for workplace design towards injury prevention in the field of ergonomics, and prosthetics design in the field of biomedical engineering and robotics. Our future experiments will involve the engagement of participants having more diverse anthropometrics, which would improve the accuracy and reliability of our findings, and add more clarity on the generation, behavior, and prediction of muscle torque.

## Data Availability

The datasets generated during and/or analyzed during the study are available from the corresponding author on request.
